# The Effect of Local Hyaluronic Acid Injection on Skin Aging: A Systematic Review and Meta‐Analysis

**DOI:** 10.1111/jocd.16760

**Published:** 2025-01-14

**Authors:** Rongying Zhou, Mei Yu

**Affiliations:** ^1^ Department of Dermatology Sichuan Huamei Zixin Medical Aesthetic Hospital Chengdu Sichuan China

**Keywords:** cosmetic, dermatology, elasticity, filler, hyaluronic acid, skin aging

## Abstract

**Background:**

Aging reduces the production of hyaluronic acid (HA) in the skin, leading to wrinkles and sagging. HA‐based skincare products are being studied to improve skin quality. This systematic review and meta‐analysis aimed to compare the effectiveness of HA‐based injectable products for reducing wrinkles and enhancing skin elasticity, hydration, and radiation.

**Methods:**

Eligible trial reports were found by searching PubMed, Embase, Scopus, and Web of Science systematically until May 2024. A random‐effects meta‐analysis was performed to evaluate the effect of HA injection on skin aging. The protocol of the study has been registered in PROSPERO with a registration ID of CRD42024540703.

**Results:**

Overall, 12 studies met the inclusion criteria, of which 6 studies had enough data for meta‐analysis. The random‐effect meta‐analysis showed improved hydration (SMD = 1.34, 95% CI = 0.14–2.54, and *p* < 0.05) and radiance (SMD = 0.51, 95% CI = 0.22–0.80, and *p* < 0.05) after HA filler injection. However, there was no significant difference in elasticity (SMD = 0.25, 95% CI = −0.20–0.70, and *p* = 0.27) and melanin index (SMD = −1.74, 95% CI = −4.89–1.41, *p* = 0.28) compared to controls.

**Conclusions:**

According to the results of a meta‐analysis, hyaluronic acid injections have been found to improve skin hydration and radiance, thus reversing the effects of skin aging. However, the study did not find any significant changes in the skin's elasticity or melanin index. Further research is required to confirm the effectiveness of hyaluronic acid in treating skin aging.

## 1 Introduction

Skin hydration and moisturization are essential aspects of maintaining healthy and youthful‐looking skin. Adequate hydration is crucial for the skin's overall health, which helps to maintain the main function as a barrier and prevent water loss and play its aesthetic role. Hyaluronic acid (HA) plays a significant role in skin hydration by retaining water molecules, thus helping to keep the skin well‐hydrated and plump.

Hyaluronic acid (HA) is a glycosaminoglycan found in many tissues throughout the body, including skin, connective tissue, and eyes. It is known for its special ability to retain large amounts of water, which makes it an essential component in maintaining skin hydration and elasticity [1, 2, 3]. HA is also a key component of the extracellular matrix, which provides structural support and plays a crucial role in maintaining skin integrity [1]. In recent years, HA has gained significant attention in the field of dermatology due to its potential benefits in improving skin appearance, particularly in reducing the appearance of facial wrinkles. HA is often used in various cosmetic products to enhance skin hydration, elasticity, and overall appearance. Despite its widespread use, the efficacy of HA in addressing specific skin concerns, such as facial wrinkles and skin hydration, remains a topic of ongoing research [4, 5].

It was said beforehand that HA is a key component of the extracellular matrix, which provides structural support and resilience to the skin. By binding to water molecules, HA helps to maintain the skin's turgor, contributing to its overall elasticity and firmness [1, 6, 7].

As we age, the natural production of HA in the skin decreases, leading to a decline in skin elasticity and the development of wrinkles and sagging. Various mechanisms have been proposed as the reasons HA injections can enhance skin properties such as hydration, radiation, elasticity, and firmness; the first one is related to the water retention capacity of HA. HA molecules can hold water many times their weight, which can help moisturize the skin and enhance the extracellular matrix, thus improving skin hydration and radiation and reducing wrinkles [8]. Another mechanism is stimulating fibroblast growth, which then enhances collagen and elastin production, which contributes to skin elasticity and firmness [8, 9]. Some studies have suggested that HA might have a depigmentation effect and also enhance the stratum corneum's barrier function, preventing transepidermal water loss even further [10]. The use of HA‐based skincare products has been explored as a potential solution to counteract these age‐related changes, with the aim of improving skin elasticity and overall skin appearance. The aim of this study is to compare the efficacy of HA‐based injectable products on skin quality.

## 2 Methods

This present study was conducted according to the Cochrane preferred reporting items for systematic reviews and meta‐analyses (PRISMA) guidelines. This study was registered with PROSPERO (registration ID: CRD42024540703).

### 2.1 Research Question

The present study examined the effect of hyaluronic acid in patients undergoing intradermal injection of HA instead of patients being injected with a placebo and only included randomized clinical trials.

### 2.2 Search Strategy and Screening

Four electronic databases (Scopus, PubMed, Web of Science, and Embase) were searched systematically until May 2024. A hand search was conducted on top of the electronic searches. Several keywords and Medical SubHeadings (MESH) terms were used in our search strategy for “hyaluronic acid,” “injection,” “elasticity,” “hydration,” “roughness,” “wrinkles,” and “aging” and modified according to the rules of databases. A detailed search strategy is available in Data S1. All records were imported using the Rayyan online systematic review software [11]. After the elimination of duplicate studies, the records were independently reviewed by two reviewers (RZ and MY), applying a distinct inclusion/exclusion criterion. Discrepancies were resolved by discussion. To mitigate publication bias, we conducted a thorough search for gray literature (unpublished reports, theses, preprints, registered trials, etc.) using Google Scholar and ClinicalTrials.gov.

### 2.3 Inclusion and Exclusion Criteria

Inclusion criteria were [1] injection of hyaluronic acid, [2] randomized clinical trials, [3] having at least two groups with one group acting as the control group with saline, placebo, or no treatment, and [4] reporting at least one primary or secondary outcome of interest (elasticity, hydration, wrinkles, and roughness).

Exclusion criteria were [1] reviews, technique articles, case reports, conference abstracts, and expert‐opinion studies, [2] studies with no control groups, [3] patients with a history of using other aesthetic methods in the last 6 months, and [4] comparison with baseline.

### 2.4 Data Extraction and Synthesis

The full text of eligible studies was reviewed, and the data was extracted using an Excel spreadsheet. First author, country, year of study, demographics, study setting, and outcomes such as wrinkles, elasticity, firmness, hydration, melanin index, and radiation were extracted. Included studies measured skin hydration using MoistureMeter devices, which measure stratum corneum hydration level; skin elasticity and firmness were measured using Cutometers; this device generates negative pressure and is put in contact with the skin; the device is then withdrawn from the skin surface, and skin elasticity is determined by measuring the skin movement. Skin radiance was assessed subjectively; the participants were advised to wash their skin completely and not apply anything on their skin for twenty minutes. Afterwards, skin radiance was measured by taking standardized photographs, and it was rated by one or a panel of blinded dermatologist(s). Mexameters were used to investigate the melanin content of skin objectively.

The rationale behind choosing these outcomes can be explained by skin aging. Skin aging can be measured using various indices: visual assessment by assessing wrinkle number, depth, and pigmentations; three‐dimensional skin imaging for the generation of standardized pictures and assessing radiance; ultrasonography and biophysical measurements using different devices like Mexameters for measuring melanin content; Cutometers for firmness and elasticity, and MoistureMeters for measuring skin hydration level. Biochemical metrics like measuring collagen and elastin levels in skin samples and measuring advanced glycation end products that accumulate in the skin during the aging process can also be used.

While visual assessments might be biased, and biochemical measurements can be less favored due to being less available and cost‐effective and possibly more invasive, biophysical properties might be the best possible metrics used for objective assessment of skincare products efficacy.

### 2.5 Quality Assessment

The JBI critical appraisal tool was used to assess the quality of observational studies (RCTs). This tool contains 13 items classified into different categories. An overall score can range from 0 to 13 based on items such as randomization, concealment, blindness, similarity, measurement, follow‐up, and study design and analysis, which are rated as yes, no, or unclear. Ratings were provided by two authors, and disagreements were resolved by discussion.

### 2.6 Statistical Analysis

Data for continuous variables were recorded as mean ± standard deviation (SD; mean and SD were used for effect size calculation). When more than one group was reported in each study, they were pooled for the analysis. The effect measure for all analyses was the mean difference. Cohen's d was used to calculate the standardized mean difference (SMD) as effect size. Cochran's Q test was used to test for heterogeneity in a study, and a *p*‐value of 0.10 was considered as evidence of heterogeneity. In addition, *I* ^2^ was used to estimate heterogeneity more accurately. For meta‐analysis, a random effects model analysis was conducted using the maximum‐likelihood method, and a *p*‐value of 0.05 or less was considered significant. If possible, meta‐regression was also performed based on the mean age of each study's participants. To evaluate publication bias in the studies, funnel plot asymmetry and Begg's test were used. The statistical analysis was conducted using the R software “meta” package.

## 3 Results

### 3.1 Study Selection

**FIGURE 1:**
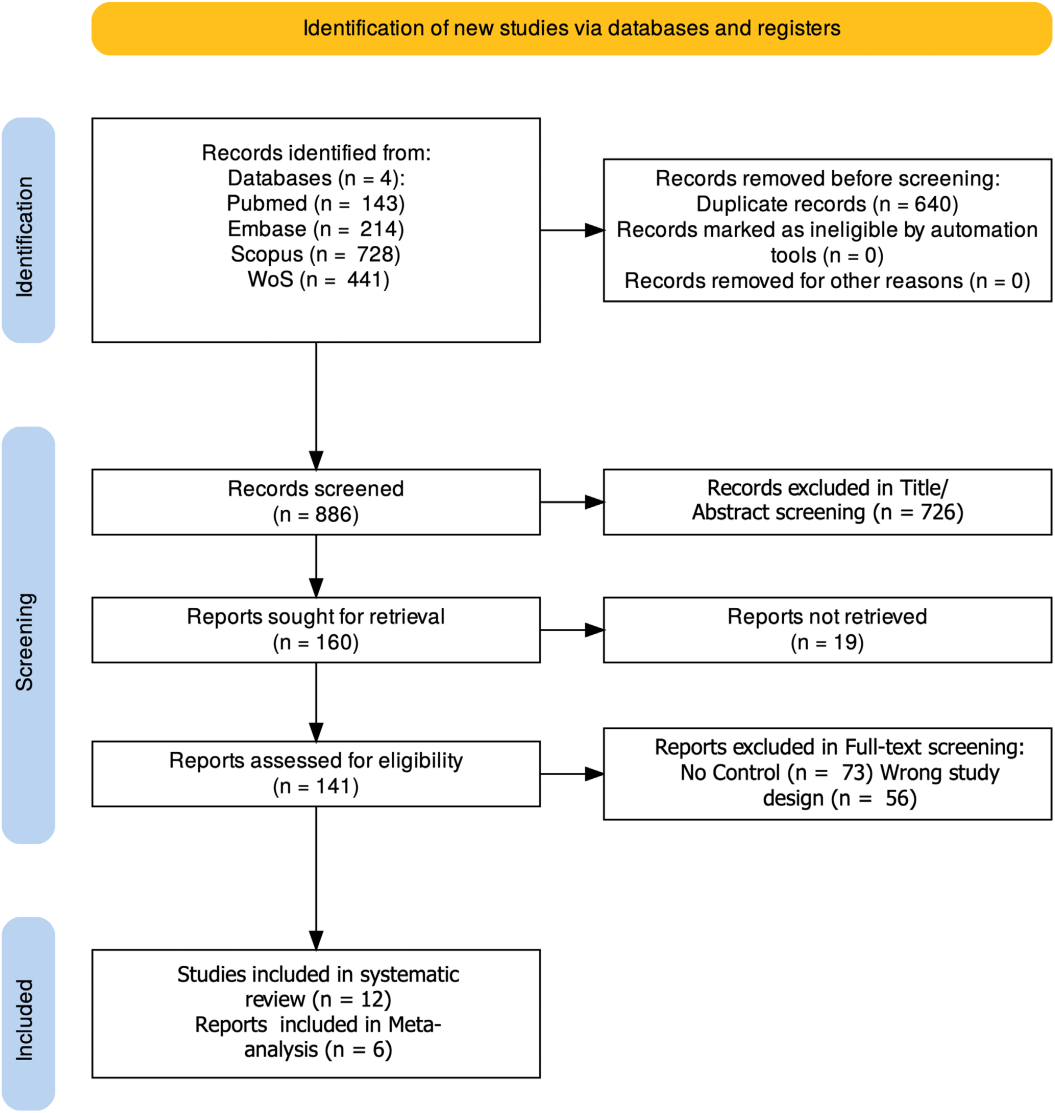
Prisma flow diagram of literature search and study selection.

The search strategy revealed 1526 results. After duplicate results were excluded, 886 results remained for screening. Title/abstract screening excluded 726 studies, leaving 160 studies for full‐text screening. After full‐text screening, 12 studies were found to be eligible to be included in the study (Figure 1) [12, 13, 14, 15, 16, 17, 18, 19, 20, 21, 22, 23]. In this phase, 73 studies were excluded due to not having control groups, and 56 studies were excluded because of having incompatible study designs or research questions with our study objectives. Six studies included enough data to perform meta‐analyses.

### 3.2 Study Characteristics and Quality Assessment

Overall, 12 studies were included in the systematic review, and 6 studies were included in the meta‐analysis [14, 15, 19, 20, 22, 23]. Overall, all studies have reported improved skin characteristics, except one that reported no improvement in skin wrinkling, elastosis, or patient satisfaction [17]. The amount of filler injection was from 0.03 to an average of 6.65 for the intervention group. Minor predicted side effects were observed in the studies, which were mainly resolved spontaneously. Only one accidental death totally unrelated to the intervention was reported [19]. The characteristics of the studies have been summarized in Table 1.

**TABLE 1:** Summary of characteristics of included studies.

First author, year	Country	No. of cases	No. of controls	Injection site	Type of filler (Amount)	Control intervention	Type of control	Overall outcome	Adverse event
Jones, 2013	USA	235	47	Face	VYC‐20 L, 6.65 mL (average)	No treatment	Other persons	Mid‐Face Volume Deficit Scale was improved. Nearly half of subjects maintained correction for 24 months.	No unpredicted adverse event occurred. Two needed treatments for their adverse events. Adverse event remained unsolved in only one subject with firmness.
Siperstein, 2022	USA	15	15	Cheek	VYC‐17.5 L, 1 mL	1 mL saline	Other cheek	Improvement in rolling atrophic scars compared to saline	No major side effect; minor side effects resolved by day 30
Siperstein, 2022	USA	20	6	Neck	HA filler Restylane Refyne, 1 mL	1 mL saline	Other persons	Significant improvement in static horizontal neck rhytides (needle was more effective than cannula)	No serious side effects
Li, 2022	USA	117	29	Lip	VYC‐15, 1.7 mL (average)	No treatment	Other persons	Lip Fullness Scale was improved based on evaluating investigators and self‐reports	One treatment‐related serious adverse event (arterial ischemia) resolved in 16 days
Fanian, 2023	France	107	38	Face and neck	NCTF135HA, 6 mL for face and 3 mL for neck	6 mL for face and 3 mL for neck anti‐aging cream	Other persons	Reduced wrinkles and improved facial radiance scores, hydration, and self‐esteem	Adverse events were mild and resolved within 48 h (48%) or a few days after injection. One accidental death
Duteil, 2023	France	28	28	Face	Non‐cross‐linked HA (M‐HA10), 3 mL	3 mL saline	Other side of face	Wrinkle depth and score decreased, and radiance, hydration, firmness, and aesthetic improved	No serious adverse event related to the study
Chiu, 2023	USA	131	74	Face	VYC‐12 L, 4 mL for both cheeks (median)	No treatment	Other persons	Significant improvement in Allergan Cheek Skin Smoothness responder rate compared to controls 1 month posttreatment.	The most common injection site reactions were redness, swelling, and lumps/bumps; most resolved within 3 days.
Jones, 2018	USA	14	14	Face	small‐particle hyaluronic acid with lidocaine, 1 mL	1 mL saline	Other side of face	No improvement in wrinkling, elastosis, or patient satisfaction	N/A
Baspeyras, 2013	France	53	53	Face	Non‐cross‐linked HA mesotherapy, 1 mL	1 mL saline	Other side of face	Non‐reticulated HA‐based mesotherapy significantly and sustainably improves skin elasticity and complexion radiance.	50 (87.7%) experienced one or several adverse effects after injection. Adverse events were generally of mild or moderate intensity and expected (hematoma, edema, papule, erythema, or other transient inflammatory reaction), and 4 experienced severe hematomas
Alexeidias, 2023	USA	131	74	Face	VYC‐12 L, 4 mL for both cheeks (median)	No treatment	Other persons	Allergan Cheek Smoothness Scale and Allergan Fine Lines Scale responder rates were statistically significantly higher in the VYC‐12 L group.	Six participants reported treatment‐related adverse events; none led to study discontinuation.
Lim, 2023	South Korea	10	10	Face	Stabilized hyaluronic acid‐based gel of nonanimal origin, 0.03	Normal saline, 0.15 mL (median volume)	Other side	All patients reported a high degree of satisfaction. The mean objective improvement score on the treated side was statistically higher than on the control side	There were no serious adverse events reported. No permanent adverse event reported.
Roh, 2016	South Korea	24	24	Face	Nonanimal stabilized hyaluronic acid, 0.18 mL	No treatment	Other side	Stratum corneum hydration was significantly improved after injection. Skin elasticity was also significantly improved during the study. The injection had no significant effect on the melanin and erythema indices throughout the follow‐up period.	The treatment was well tolerated, and no serious adverse events were reported.

*Note:* The risk of bias assessment demonstrated that all studies had less than 50% risk of bias, but no one had 0% risk of bias. The detailed quality assessment for the included studies is shown in Table 2.

**TABLE 2:** Summary of quality assessment for included studies.

First author, year	#1	#2	#3	#4	#5	#6	#7	#8	#9	#10	#11	#12	#13	Overall study quality^a^
Jones, 2013	N	Y	Y	Y	Y	Y	Y	Y	Y	Y	Y	Y	Y	92%
Siperstein, 2022	N	Y	Y	Y	Y	N	Y	Y	Y	Y	Y	Y	Y	85%
Siperstein, 2022	Y	Y	Y	Y	N	Y	Y	Y	Y	Y	Y	Y	Y	92%
Li, 2022	N	Y	Y	Y	N	Y	Y	Y	Y	Y	Y	Y	Y	85%
Fanian, 2023	N	N	N	Y	N	Y	Y	Y	Y	y	Y	Y	y	69%
Duteil, 2023	N	Y	N	N	N	Y	N	Y	Y	Y	y	y	y	62%
Chiu, 2023	Y	N	N	N	N	Y	Y	Y	Y	Y	Y	Y	Y	69%
Jones, 2018	N	N	Y	Y	N	Y	Y	Y	Y	Y	Y	Y	Y	77%
Baspeyras, 2013	N	N	Y	Y	N	Y	Y	Y	Y	Y	Y	Y	Y	77%
Alexeidias, 2023	Y	N	Y	N	N	Y	Y	Y	Y	Y	Y	Y	Y	77%
Lim, 2023	N	N	Y	N	N	Y	Y	Y	Y	Y	Y	Y	Y	69%
Roh, 2016	N	N	Y	N	N	Y	Y	Y	Y	Y	Y	Y	Y	69%

a The percentage indicates the overall quality of the study. A higher percentage reflects better study quality and a lower risk of bias.

### 3.3 Hydration

**FIGURE 2:**
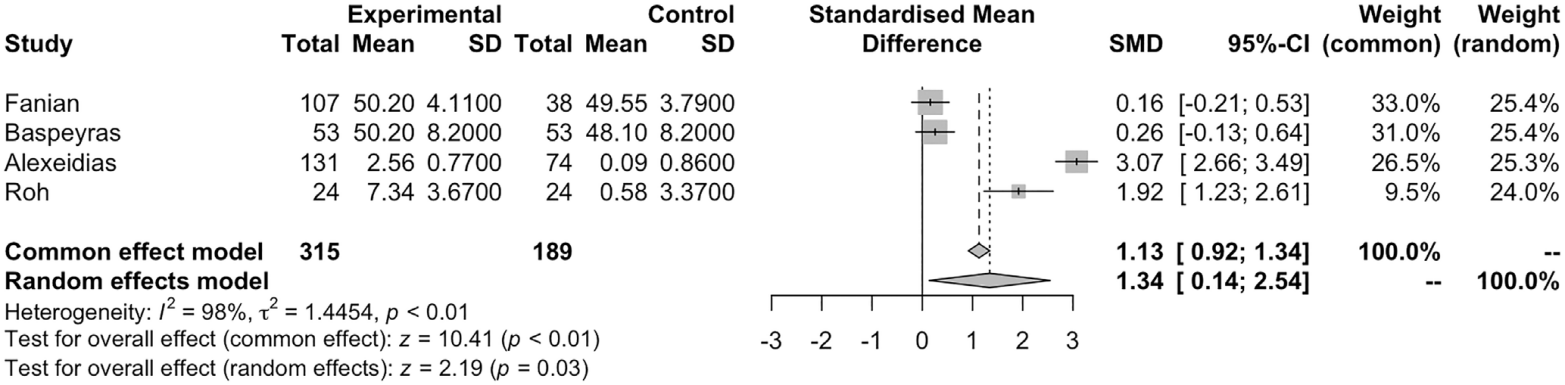
Forest plot for the hydration meta‐analysis.

Four studies with an overall 404 subjects were included in the meta‐analysis. Among them, 315 received HA‐based fillers, and 189 received non‐HA‐based fillers as a control. The random‐effect meta‐analysis demonstrated a higher hydration score in the HA‐filler group compared to controls (SMD = 1.34, 95% CI = 0.14–2.54, and *p* < 0.05; Figure 2). The heterogeneity of the studies was significant, with *I* ^2^ = 98%, tau^2^ = 1.45, and *p* < 0.05. It was observed that there was no publication bias among the studies (*p* = 0.17). Meta‐regression demonstrated that age does not significantly affect the result (*p* = 0.99). The sensitivity analysis showed that Alexeidias is potentially an outlier in the analysis. Repeating the analysis after removing Alexeidias led to an SMD of 0.73 (95% CI = −0.14–1.59 and *p* = 0.10).

### 3.4 Elasticity

**FIGURE 3:**
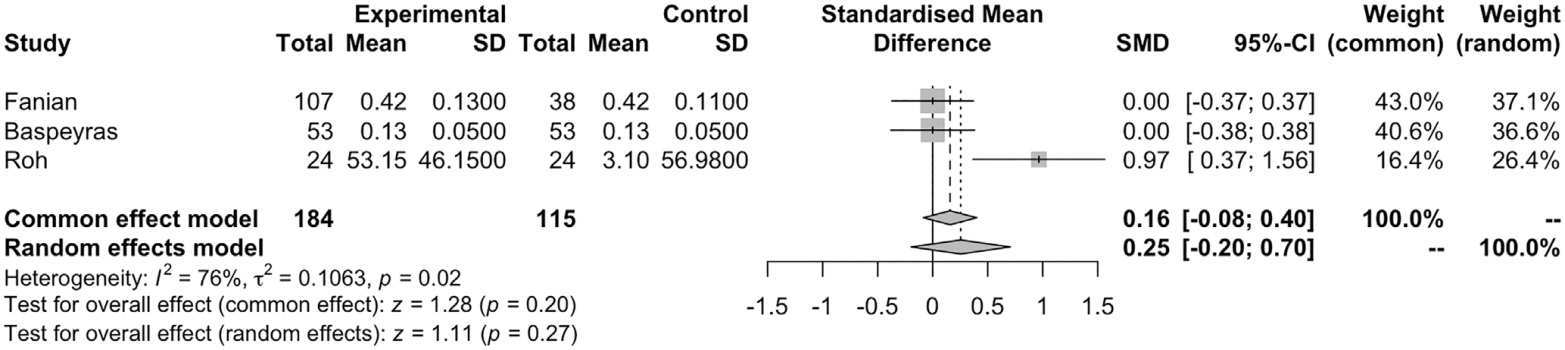
Forest plot for the elasticity meta‐analysis.

Three studies with 299 subjects (184 in intervention and 115 in control groups) were included in the meta‐analysis. The random‐effect meta‐analysis demonstrated a non‐significant improvement in the elasticity of the skin (SMD = 0.25, 95% CI = −0.20–0.70, and *p* = 0.27; Figure 3). The heterogeneity of the studies was significant (*p* < 0.05, tau^2^ = 0.11, and *I* ^2^ = 76%). Publication bias among studies was not significant (*p* = 0.11). Meta‐regression showed an inverse association between the age of the participants and elasticity, with the lower age having higher skin elasticity (*p* < 0.05). The sensitivity analysis detected no outliers for this analysis. The lack of significant improvement in elasticity may be due to the fact that elasticity is influenced by more profound structural components.

### 3.5 Radiance

**FIGURE 4:**
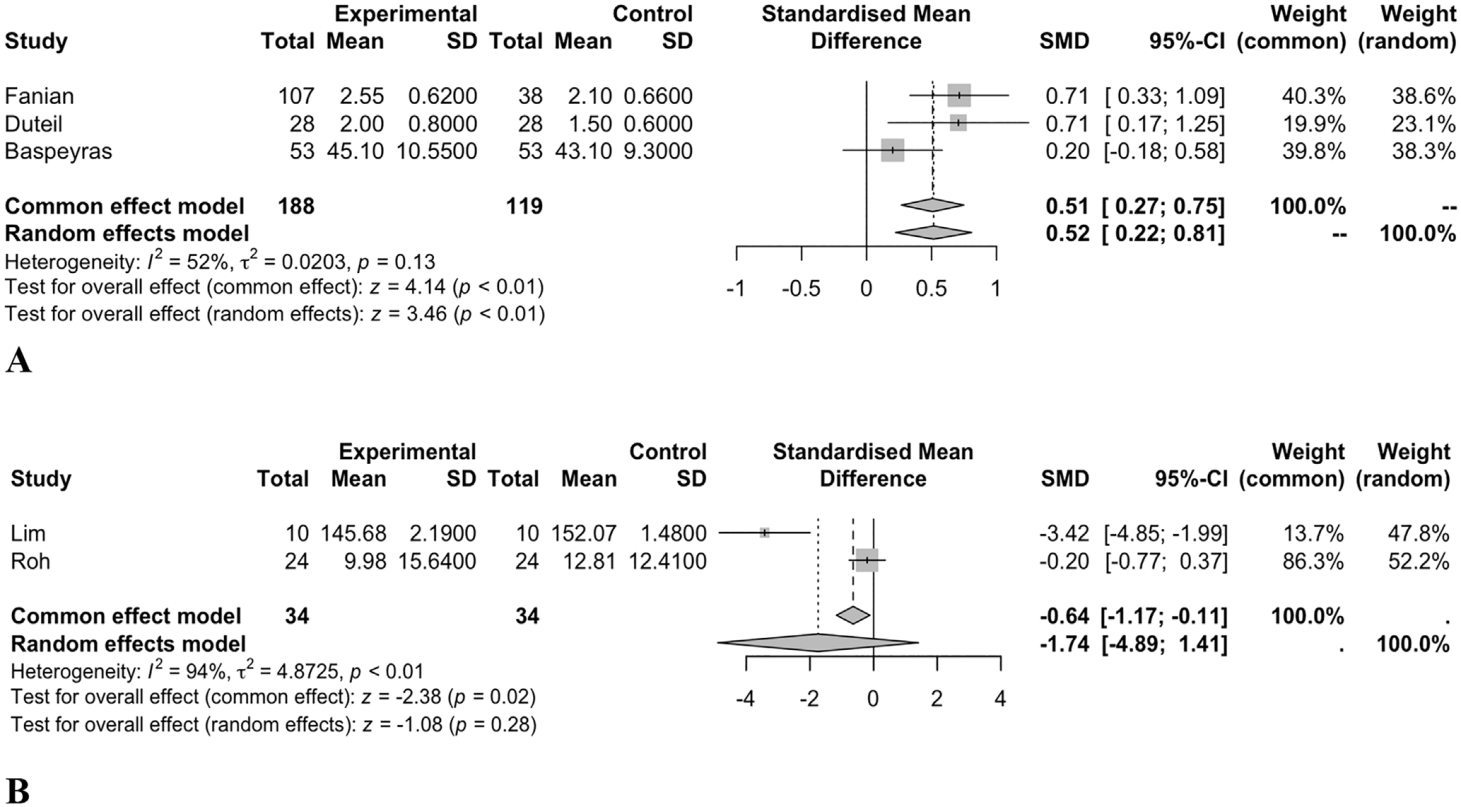
(A) Forest plot for radiance meta‐analysis. (B) Forest plot for melanin index meta‐analysis.

Three studies with 307 participants overall (188 in intervention and 119 in control groups) were included in the meta‐analysis. Random‐effect meta‐analysis showed a significant improvement in the radiance of the skin following HA filler injection compared to control (SMD = 0.51, 95% CI = 0.22–0.80, and *p* < 0.05; Figure 4A). The heterogeneity of the studies was not significant (*p* = 0.11, tau^2^ = 0.02, and *I* ^2^ = 50.8%). The publication bias was not significant (*p* = 0.60). Meta‐regression demonstrated that age did not have a significant association with the skin radiance following skin HA‐filler injection (*p* = 0.13). The sensitivity analysis detected no outliers.

### 3.6 Melanin Index

Two studies with 68 participants (34 in intervention and 34 in control groups) were included in the meta‐analysis. Random‐effect meta‐analysis demonstrated that the reduction in melanin index was not significant following the HA‐filler injection (SMD = −1.74, 95% CI = −4.89–1.41, *p* = 0.28; Figure 4B). The heterogeneity of the studies was significant, with *p* < 0.001, tau^2^ = 4.87, and *I* ^2^ = 94.1. Publication bias and meta‐regression were not applicable due to the low number of studies. No outliers were detected in the random‐effects meta‐analysis during the sensitivity analysis. The lack of a significant effect of HA on the melanin index may be attributed to the fact that melanin production is influenced by genetic factors and external environmental exposures, such as UV radiation, which may not be directly affected by HA filler injections.

## 4 Discussion

Overall, our study results showed that HA‐based injections did produce a significant effect on the radiance and hydration of participant skins, and the changes in elasticity and melanin index were not significant.

Throughout different studies, HA has been studied and has been shown to have a positive effect on skin quality measured by different metrics. However, there is a huge variability between study designs since administered HA varies by molecular weight and density, and nowadays, many HA fillers use crosslinking technology in order to improve longevity. Some HA fillers also include lidocaine for anesthetic purposes. Some studies used dilution techniques with saline, and some other studies administered oral HA supplements.

HA can contribute to skin rejuvenation by different mechanisms; HA fillers have a physical filling effect that helps in cases of severe facial wrinkles simply by physically filling them up, giving them a youthful appearance [9]. Another mechanism by which HA fillers help with facial rejuvenation is improving skin hydration, as HA molecules can retain water and, thus, can help in skin hydration and wrinkle reduction by that mechanism [1, 14, 17, 19, 20, 21, 22, 23]. Another mechanism is collagen production; HA can stimulate collagen production, leading to better skin elasticity and fewer wrinkles with less depth [20]. The other mechanism by which HA can help skin quality augmentation is its anti‐inflammatory properties. When combined with anesthetics such as lidocaine, it has been observed that post‐injection swelling and redness were further decreased [16, 17]. Some studies have also investigated other variables such as wrinkle depth, skin thickness, patient satisfaction, luminosity, firmness, and scarring in HA filler‐injected patients versus controls [12, 13, 14, 15, 16, 17, 18, 19, 20, 21, 22, 23]. A study conducted by Betemps et al. showed that HA fillers improved elastin, collagen type I, collagen type III, and fibrillin‐1 expression in the injection site [24]. However, our study results did not show a significant difference for elasticity and melanin index. This can have various reasons considering the mechanism by which HA is known to affect skin elasticity and melanin index; skin elasticity is mainly determined by the amount and structure of skin collagen and elastin fibers. The main role of HA injections is to enhance skin hydration and create intradermal tension, indirectly improving collagen and elastin production and extracellular matrix enhancement. This indirect effect can depend on many factors, such as the injection technique, structural changes of skin due to aging, dietary and nutritional aspects of patients' lifestyles, the amount and concentration of HA injected, and the variations between different types of HA used. Regarding melanin index reduction, although some studies have suggested that HA can lead to a reduction in melanin index when combined with other products, no exact mechanism is known as of today by which HA can affect melanocytes [10]. Melanin index can also differ greatly between individuals due to genetic factors, variations in skin types, and existing melanocyte activities. This means that further studies accounting for intergroup variations have to be conducted in order to find, if any, possible significant relationship between melanin index and HA. The differences in the impact on elasticity versus hydration and radiance likely stem from distinct biological mechanisms. Hydration affects skin plumpness and surface texture, while elasticity is influenced by deeper components like collagen and elastin fibers. This highlights the complexity of skin's response to HA fillers, showing that while they effectively enhance hydration and radiance, their impact on elasticity may be more variable.

However, HA fillers can also have some side effects. These can be summarized as redness, pain, tenderness, firmness, swelling, itching and bruising, bumps, and rarely discoloration. This necessitates careful administration and follow‐up and patients undergoing HA filler injections for any minor and major side effects.

Our study was limited by the scarcity of studies that were included since our inclusion criteria necessitated that the included study compared HA fillers with placebo or saline injections, and there had to be a control arm in the study. The long‐term efficacy of HA fillers is a matter that can be addressed in future studies.

## 5 Conclusion

HA‐based fillers, when used for the right patients and with the right dosing and timing, can contribute to skin quality when compared to controls. Radiance and hydration of participant skins were significantly improved when compared to control subjects. The elasticity change was not significant compared to controls.

## Conflicts of Interest

The authors declare no conflicts of interest.

## Supporting information


Data S1.

## Data Availability

The data that support the findings of this study are available from the corresponding author upon reasonable request.
