# Supplementary material for: The Effect of Local Hyaluronic Acid Injection on Skin Aging: A Systematic Review and Meta‐Analysis
Source: J Cosmet Dermatol. 2025 Jan 14;24(1):e16760. doi: 10.1111/jocd.16760 (PMC11731322; doi:10.1111/jocd.16760)
Supplement: Supplementary file 1 — Data S1. [file JOCD-24-e16760-s001.docx]

**PubMed:**

("hyaluronic acid") AND

("injection" OR "filler") AND

("aging" OR "ageing" OR "wrinkle*" OR "Rough*" OR "elast*" OR "hydra" OR "rhytid*")

**Scopus:**

TITLE-ABS("hyaluronic acid") AND

TITLE-ABS("injection" OR "filler") AND

TITLE-ABS("aging" OR "ageing" OR "wrinkle*" OR "Rough*" OR "elast*" OR "hydra" OR "rhytid*")

**Web of Science:**

TS=("hyaluronic acid") AND

TS=("injection" OR "filler") AND

TS=("aging" OR "ageing" OR "wrinkle*" OR "Rough*" OR "elast*" OR "hydra" OR "rhytid*")

**Embase:**

(‘hyaluronic acid’:ti,ab) AND

(‘injection’:ti,ab OR ‘filler’:ti,ab) AND

(‘aging’:ti,ab OR ‘ageing’:ti,ab OR ‘wrinkle*’:ti,ab OR ‘Rough*’:ti,ab OR ‘elast*’:ti,ab OR ‘hydra’:ti,ab OR ‘rhytid*’:ti,ab)
